# On the Treatment of Fibrous Tumors of the Uterus

**Published:** 1865-04

**Authors:** C. H. F. Routh

**Affiliations:** Physician to the Samaritan Hospital for Women and Children


### ON THE TREATMENT OF FIBROUS TUMOURS OF THE UTERUS.

By Dr. C. H. F. ROUTH, Physician to the Samaritan Hospital for Women and Children.

[The surgical treatment of Fibrous Tumours of the Uterus is of two kinds: 1. Enucleation of the Tumours. 2. Removal of the tumour by gastrotomy. Enucleation may be primary where it is completed at the time or within a day or two, or it may be effected by inducing gangrene or death of the tumour by sloughing. In 1857, Mr. Hutchinson published some papers on this subject. (*Retrospect*, vol. xxxvi., p. 241.)

The principal rules laid down by Mr. Hutchinson are: "1. The tumour must be well depressed into the pelvis by an assistant. 2. The first incisions must be very free, and pass deeply down into the tumour; thus not only completely dividing the capsule, but facilitating its bisection, should that afterwards be found requisite. 3. The opened capsule must be separated by the fingers, or, if needful, by blunt-pointed scissors, the finger being used as a director. Strong and large vulsella, with midwifery forceps, should be at hand, to be used for traction, if necessary. 4. The grand object is to draw down, after separation of the tumour, the uterus inverted with the tumour to the external parts, or as near as possible to them, which facilitates the operation. 5. After eversion has been completed, an examination should be made with the finger, also *per rectum*, &c., so as not to cut through an inverted pouch of peritoneum in separating the final attachments of the uterine tumour. 6. The uterus is then returned. Ice, ergot, stimuli, &c., are to be given as indicated by the symptoms."

The views here epitomised are so very much in accordance with common sense and sound surgery that I do not wish as a whole to controvert them. To one point only of these I would take exception. I do not believe that the grand object is to draw down the inverted uterus after separating the tumour, so as to bring the tumour as near as possible to the external parts, because this renders the operation easier.

It cannot be doubted that simple traction of the uterus will often suffice to determine inflammation in and about the peritoneum around the organ. In the fatal cases of enucleation, death seems to have been due to phlebitis or peritonitis. Upon this practical point we can learn a lesson from the experience of practitioners in the removal of *polypi*, as a rule, a far easier operation if the uterus be pulled down.

One case is mentioned by M. Gaubrie (*Bull. de la Soc. Anat.*, vol. xvii., p. 209) where a polypus, supposed to be attached to the cervix (but found after death to be attached to the fundus), was pulled down forcibly. The pedicle yielded and it came away; yet that patient died from latent peritonitis. After death, the uterus was found healthy; but pus was found in the pelvis. Another case is mentioned by M. Demeaux (*Ibid.*, vol. xviii., p. 41) in which an enormous polypoid growth filled the cavity of the uterus. It was pulled down to the vulva, removed, and the parts replaced. There was no bleeding. Peritonitis followed and death two days subsequently. The *post mortem* examination revealed pus in the pelvic cavity. No uterus could be found in the pelvis; but a puckering and a sunken-in portion, within which the ovaries and a vaginal tumour, which was the inverted uterus, were found. This inversion, it is true, was supposed to have been the result of the weighty polypoid growth before traction had been employed. Yet death from peritonitis was the result of the operation. A more remarkable case, however, is quoted by M. Pigné (*Ibid.*, vol. xiv., p. 11). This was a polypus as large as a fist, which was taken from a dead woman. During life an attempt was made to remove it; but the finger being first introduced within the rectum, no uterus could be felt. All extraction was now stopped, on the supposition that the tumour was an inverted uterus. Peritonitis and death followed. The *post mortem* examination revealed a pediculated polypus within a very small and anteverted uterus. No injury whatever of the peritoreum had taken place. The traction alone produced the peritonitis. Dr. Greenhalgh has informed me of a case which he saw at Würzburg, where the mere traction of the uterus downwards produced peritonitis and death.

In the recorded cases of enucleation, where traction is mentioned particularly as having been made, the results may be referred to two heads; viz., where the uterus or the tumour was pulled down by fingers, a string, or other violent means; and, secondly, where this was done by forceps. The result in the two cases is very different. Of eight cases, in all but two of which the operation was that of primary enucleation—one being secondary, the other failing altogether—in three, the uterus was for the time completely inverted. Three cases died; in one of these, eversion had occurred; in another, forcible attempts were made to pull down the uterus, which failed.

In eight other cases, in all of which but one (where the operation failed, although the tumour sloughed away afterwards) the operation was primary enucleation, and the forceps were used, no deaths occurred. In two, the uterus was inverted. In two cases, it was the uterus which is stated to have been pulled down by the forceps. In the remaining six, it was the tumour which was so drawn down. All did well, except one patient, who had phlegmasia, and whose convalescence was not established till two months. All the tumours, however, where it is stated, were small; except in the patient who had phlegmasia, where it was large; and in another, where the tumour weighed eighteen ounces, and the diameters were four inches by six.

Was this difference in the result due to the better regulated traction which can be exerted by a forceps, or to the better direction given by it? Probably, where the forceps were used, the tumours were low down in the true pelvis, and so easily included in them. Short forceps were used in every case; and, therefore, it would seem that the traction was less forcible. Whenever these were used, the results were more satisfactory. The fact is indubitable. Still it seems difficult to say to what cause the occurrence of peritonitis is due, where it so occurs; when we know that in very many other cases where traction is employed it does not occur.

In explanation, I have but three suggestions to make. In cases of retroversion, I have never seen peritonitis follow traction to restore the organ to a straight direction, so as to allow the admission of the sound. Is it because in these cases the peritoneum is no longer in its normal state, already changed by the very unusual disturbances to which it has for some time past been subject? This is certainly true in many cases of ovariotomy, where numerous adhesions exist. Secondly, peritonitis, and accidents of like nature, are rare, if our patients have been well purged and prepared by remedies before operation; a precaution, which, in the case of polypoid growths, is often neglected. Will this explain the difference? Thirdly, there is doubtless, in some cases, idiosyncracy and a predisposition to peritonitis.

The practical conclusion, at any rate, to be deduced is, that the very traction which is necessary in these cases is often in itself a source of danger and a cause of death; and, therefore, if possible, it should be avoided.

*Enucleation by the Induction of Gangrene*, or *Secondary Enucleation*, as it is called, is a much more tedious operation. Out of fifteen cases mentioned by Mr. Hutchinson, nine recovered, or 60 per cent.; and six died, or 40 per cent. In my table, of ten cases, in one the result was not stated. Of the remaining nine, four died, or 44.4 per cent.; and five recovered, or 55.6 per cent.

Mr. Hutchinson has concluded that, if other unpublished cases were taken into account, the results of the two modes of operation would be identical. The above figures, however, so far as they go, prove that enucleation by the induction of gangrene is positively less fatal than primary enucleation. Is this due to the traction which is employed being less marked?

The modes in which this operation has been practised have been various. The French method consists in using the knife, in most cases without the use of ergot at all. Dr. Atlee's consists in using, in the first place, ergot in repeated doses, so as to influence the uterus to contract and forcibly eject the tumour. Then the incision is made through the capsule, the ergot being continued, and the tumour gradually separated from the cyst by the finger, cutting, by the knife or scissors, any adhesions which may interfere with such separation. This is continued from time to time; the tumour in the meanwhile sphacelates and comes out by pieces, until the whole has come away, or what remains is capable of enucleation and removal. There can be no doubt that Dr. Atlee's method is an improvement upon that of the French school. It provides a *vis a tergo* to assist the operator.

Again, Dr. Atlee does not limit his operation of enucleation to the *uterus*. In several cases where, from the size of the tumour or its position low down in the pelvis, while the uterus is high up or difficult to reach, and in cases of extrauterine fibroid, which occupies, similarly a true pelvic position, he does not hesitate to cut right through the *vagina*, so enucleating the tumour. This he performed in several cases; and, so far as I know, he was the first to attempt this mode of procedure.

In estimating the degree of mortality due to enucleation by the induction of gangrene, we learn again a lesson from what occurs in some cases of polypi, which are tied and allowed to slough away.

The experience of Drs. Robert Lee and M'Clintock gives the following numerical results of cases in which the ligature was applied:—

| | Cases. | Deaths. | Per Cent. |
|---|---|---|---|
| Dr. Robert Lee | 20 | 9 | 45 |
| Dr. M'Clintock | 10 | 3 | 30 |
| | 30 | 12 | 40 |

And, no doubt, with a large sloughing mass in the vagina, exposed to the air and putrefaction, the absorption of putrid pus is a natural result. The same is true with regard to the fibroid within its cyst, only to a greater degree. In the case of polypus, we have merely mucous membrane, which is our absorptive surface. In the case of the enucleated fibroid, we have a raw ulcerated surface of the cyst, that part which is the vascular part of the tumour, and therefore eminently absorptive, ready to take into the system the vitiated pus. The surprise is that, in such cases recovery ever occurs. Fortunately, the *vis medicatrix naturæ* is often powerful enough to accomplish it. It is for this reason that I think Mr. Brown overrates the beneficial results of this modification in the operation, in first incising the os and allowing it to heal before he proceeds to gouging or enucleation. It is of use, doubtless, as it removes one additional source of absorption; but others remain.

The practical lesson which is taught by these remarks, is the necessity of frequent injections and washing with disinfectants, and obviating the production and long contiguity with ulcerated surfaces of effete and putrid matters.

*Enucleation by Gouging*, and inducing gangrene subsequently, is a modification of Mr. Brown's.

The instrument which he uses I now show you. It has already been exhibited before this society, and, therefore, need scarcely detain us long. It was originally devised by Mr. Philip Harper. It consists of a hollow tube of steel with cutting knives. Contained in this tube is a hook, which can be pushed up by a spring, and thus grasps the tumour, whilst circular knives are carried through by means of a screw. In this way, a piece can be actually cut out of a tumour much in the same way as the central piece is cored out of an apple.

In the *Obstetrical Transactions*, Mr. Brown has published several cases exemplifying this mode of treatment. I may remark, however, that it is restricted by him to *intrauterine fibrous tumours of the non-pedunculated form*, growing from the inside of the uterus from a broad base. Mr. Brown has given us four cases in which he gouged in this manner. In three, a cure followed; but in one, the operation led to a fatal result. This death he attributes to the absorption of putrid pus through the cut edges of the cervex, which was previously laid open to expose the contained tumours. Hence, at present, his mode is in the first place to lay open the cervex, and to wait two or three days or longer, till this has healed, before he proceeds to gouge.

The method pursued after the gouging, is to plug up the hole thus made by oiled lint, to arrest hemorrhage and provoke sloughing of the tumour.

In three of his published cases, however, the tumours were not gouged. In one, the tumour was broken up by a pair of sharp scissors.

I may say that through the kindness of Mr. Brown, (who has given me the notes of several of his cases, even hitherto unpublished), I know of two more examples in which, after the incision of the os, the tumour was broken up by scissors; in all of which recovery, in one case disappearance of the tumour, followed.

But a method which is likely to supersede in great measure these more bloody operations, is simple incision of the os, subsequently carried right through a portion of the tumour.

A published case of Mr. Brown, given in the *Obstetrical Transactions*, (vol. iii., p. 76,) is, however, peculiarly interesting, as the cure was open to ocular inspection, a piece of good fortune not always met with.

This was the case of a single woman, in whom, some seven years previously, he had removed from the os uteri a fibrous growth of about the size of a walnut; and in whom, at the time of the second operation, he had distinctly made out three fibrous tumours just within the os and projecting into the vagina. The os and cervix were freely cut open; and then each of these tumours was deeply cut into; the cut surfaces being dressed with oiled lint, and the vagina plugged. All these dressings were removed in forty-eight hours, and the vagina daily injected. A month afterwards, two out of the three growths had entirely disappeared, and the third was reduced to half its size.

In two other unpublished cases furnished to me by Mr. Brown, the same result was as conclusively observed. I do not dwell on these, because they are as yet unpublished. Altogether, he has mentioned to me ten cases in which this simple plan of incision of the external os was practised with the best results. As these are, however, only a few among several which will shortly be made public, I only ask you to wait and judge for yourselves.

Here we have, then, a practical lesson taught us. The mere incision will suffice to cause the absorption of the tumour. It is a lesson thus taught, from which Mr. Brown appears himself to have profited, as I now learn that in most cases he no longer gouges, but makes free incisions. The plan is safer. Sloughing in the tumour is thus set up, and this is disintegrated, and diminishes in size, and finally disappears.—*British Medical Journal*, *July* 16, 1864, *p.* 53.

